# Impact of amlodipine on clinical outcomes for heart failure in patients with dilated cardiomyopathy: a Korean nationwide cohort study

**DOI:** 10.3389/fcvm.2023.1305824

**Published:** 2023-11-17

**Authors:** SungA Bae, Wan Kee Kim, Seng Chan You, Minkwan Kim, In Hyun Jung

**Affiliations:** ^1^ Department of Cardiology, Yongin Severance Hospital, Yonsei University College of Medicine, Yongin, Republic of Korea; ^2^ Department of Thoracic and Cardiovascular Surgery, Hanyang University Seoul Hospital, Hanyang University College of Medicine, Seoul, Republic of Korea; ^3^ Department of Biomedical Systems Informatics, Yonsei University College of Medicine, Seoul, Republic of Korea

**Keywords:** dilated cardiomyopathy, heart failure, amlodipine, all-cause death, hospitalization, hypertension age, sex, hypertension

## Abstract

**Introduction:**

Amlodipine, widely used as a first-line treatment for hypertension, has inconclusive clinical evidence regarding its efficacy in patients with heart failure. This retrospective cohort study aimed to investigate the clinical effectiveness of amlodipine treatment after hospitalization for heart failure in patients with dilated cardiomyopathy (DCMP).

**Methods:**

A total of 20,851 patients who were diagnosed with DCMP and admitted for heart failure between 2005 and 2016 according to Korean nationwide medical insurance service database were enrolled. Amlodipine use was defined as its prescription at the time of discharge and for at least 180 days within a year. The primary outcome was all-cause death, and the secondary outcome was heart failure rehospitalization during a 5-year period. The outcomes between patients who received amlodipine (*n *= 6,798) and those who did not (*n *= 14,053) were compared.

**Results:**

During the 5-year follow-up, the group treated with amlodipine exhibited a significantly lower risk of all-cause death and heart failure rehospitalization than the group not treated with amlodipine [all-cause death: adjusted hazard ratio (HR): 0.64, 95% confidence interval (CI): 0.59–0.70, *p* < 0.001; cardiovascular death: adjusted HR: 0.71, 95% CI: 0.62–0.81, *p* < 0.001; heart failure rehospitalization: adjusted HR: 0.92, 95% CI: 0.86–0.98, *p *= 0.006]. In a subgroup analysis, amlodipine had a significant impact on decreasing all-cause mortality in older adults, those with a higher systolic blood pressure, and those with a lower Charlson Comorbidity Index.

**Conclusion:**

In summary, amlodipine use after hospitalization for heart failure in patients with DCMP was associated with a lower risk of all-cause death and readmission for heart failure.

## 1. Introduction

Heart failure (HF) is a major public health issue that affects millions of individuals worldwide, with its prevalence almost doubling from 33.5 million in 1990 to 64.3 million in 2017 (1). Despite advancements in medical therapy, HF remains a major cause of morbidity and mortality globally (2). One major concern with calcium channel blockers, commonly used medications for hypertension and angina, is their potential to worsen HF and increase the risk of death, particularly in patients with advanced left ventricular (LV) dysfunction (3–5). Consequently, physicians are advised to avoid prescribing calcium channel blockers to patients with HF, even when treating coexisting angina or hypertension (6).

Amlodipine, a long-acting calcium channel blocker, has been demonstrated to have fewer adverse effects than other agents (7, 8). Although amlodipine has been shown to be safe and effective in treating hypertension and angina, evidence of its effectiveness in patients with HF and non-ischemic cardiomyopathy is still inconclusive (9). Previous studies have yielded inconsistent clinical outcomes. The Prospective Randomised Amlodipine Survival Evaluation (PRAISE) 1 trial revealed that patients with severe chronic HF and non-ischemic cardiomyopathy who were taking amlodipine had a lower risk of death than those who were taking a placebo (10). However, the subsequent PRAISE 2 trial did not find any clinical benefits of amlodipine (11). Given the inconsistent outcomes shown in previous studies, the effectiveness of long-term amlodipine treatment in patients with HF remains unclear. Therefore, we aimed to investigate the clinical efficacy of amlodipine treatment after hospitalization for HF in patients with dilated cardiomyopathy (DCMP), using the latest nationwide medical insurance data from the Korean population.

## 2. Methods

This was a retrospective cohort study using a database from the National Health Insurance Service (NHIS) in South Korea, we identified patients with HF and DCMP based on codes of the International Classification of Disease-10th Revision-Clinical Modification (ICD-10-CM) system. The NHIS is an obligatory health insurance service in South Korea that provides medical coverage for most of the Korean population, except for low-income group (approximately 3%, who are covered by the Medical Aid program). The database includes personal profiles and medical information, such as diagnoses, treatments, and death records. Diagnoses and treatments (whether medical or procedural) were documented using ICD-10-CM codes. These data are accessible only to government facilities. The present study was waived from ethical review by the appropriate institutional review board. The investigation was conducted in accordance with the principles outlined in the Declaration of Helsinki.

### 2.1. Study population

First, we identified 100,566 patients who had previously been diagnosed with DCMP based on the ICD-10-CM codes (I42) between 2005 and 2016. The diagnosis of DCMP was established based on the following criteria: (i) presence of LV dilation (LV end-diastolic diameter ≥55 mm); (ii) presence of reduced LV ejection fraction (all ≤45%); (iii) coronary angiographic evidence of the absence of coronary artery disease defined as >50% stenosis of a major epicardial vessel or a history of myocardial infarction; and (iv) absence of cardiac muscle disease secondary to any known systemic disease (12). We excluded patients who had no hospitalization for HF (*n *=* *51,325), were aged <20 years (*n *=* *1,024), had a history of myocardial infarction or percutaneous coronary intervention (*n *=* *4,413), had been prescribed other calcium channel blockers (*n *=* *12,595), had used amlodipine types within 6 months prior to the index HF event (*n *=* *9,552), or died within 30 days after the index HF event (*n *=* *806). Amlodipine use was defined as its prescription at the time of discharge and for at least 180 days within a year.

### 2.2. Clinical outcomes and covariates

The primary outcome of this study was designed to assess the difference in all-cause mortality over a 5-year period between the amlodipine user and non-user groups. In addition, cardiovascular mortality information was recorded. Data on mortality were sourced from the integrated clinical data server analysis system and the National Statistical Office of Korea. Patient follow-ups continued until either all-cause death or 31 December 2021, the latter marking the last recorded date of the patient's survival by the National Statistical Office of Korea.

The secondary outcome was focused on evaluating the disparities in heart failure (HF) rehospitalization rates between two groups. HF rehospitalization was defined as a new I50 diagnostic code assigned upon admission after an initial HF diagnostic code was established. The study participants were followed up until either the occurrence of each endpoint or 31 December 2021, whichever occurred first.

Detailed definitions of comorbidities such as hypertension, diabetes mellitus, dyslipidemia, valvular heart disease, atrial fibrillation, chronic kidney disease, and malignancy are provided in Supplementary Table S1. The Charlson Comorbidity Index (CCI) was also calculated as previously described (13). We examined the medication history of patients, which included data on renin–angiotensin–aldosterone system inhibitors (such as angiotensin-converting enzyme inhibitors or angiotensin receptor blockers), beta-blockers, spironolactone, other diuretics (loop or thiazide series), digoxin, statins, antiplatelet agents, and anticoagulants.

### 2.3. Statistical analysis

Descriptive statistics are used to present the mean ± standard deviation for continuous variables and numbers (percentages) for categorical variables. Unpaired Student's *t*-test was used for continuous variables, and the *χ*^2^ test or Fisher's exact test was used for categorical variables to compare groups, as appropriate. Kaplan–Meier curves were employed to establish the cumulative incidence of primary and individual outcomes using a log-rank test.

Cox proportional hazard regression analyses were performed to calculate the hazard ratio (HR) with a 95% confidence interval (CI) for the primary and secondary outcome between the amlodipine user and non-user groups. Because differences in the baseline characteristics of patients could have a significant impact on clinical outcomes, sensitivity analyses were performed to account for confounding factors as much as possible. First, a Cox proportional hazards regression model was used to evaluate the clinical outcomes with adjustments for covariates, and variables in the multivariate analysis were selected based on their significance levels (*p* < 0.1) or predictive values. Second, propensity score matching was conducted between the two groups, with propensity scores obtained from logistic regression, including demographics, procedural characteristics, and medication at discharge, as covariates. Using nearest-neighbor matching with linearly transformed propensity scores (logit transformation), 2,194 patients in the amlodipine group were matched with 2,194 patients in the no-amlodipine group. The standardized mean difference after propensity score matching was ≤10% across all matched covariates, demonstrating successful balance achievement between the comparison groups. Third, we conducted an adjustment using inverse probability weighting by assessing the inverse propensity score of all variables using a proportional hazard regression model. Fourth, to select a group that meets more stringent diagnostic criteria for DCMP, we conducted an additional analysis focusing on patients who satisfy two specific conditions: (1) a diagnosis by the ICD-10 code of I42, and (2) eligibility under the Rare Intractable Disease (RID) code-based diagnosis (V127). Patients registered under the RID receive special medical aid, with the NHIS covering 90% of their medical expenses. Due to these substantial benefits, their diagnosis and condition are rigorously validated and monitored through detailed clinical and imaging evaluations. Regular assessments are conducted by a team of healthcare experts and insurance specialists, adhering strictly to the guidelines established by the Ministry of Health and Welfare. Fifth, we conducted a “falsification endpoint” to assess the potential for systematic bias in the study, using 13 pre-specified falsification endpoints with true HRs of 1 (14).

All statistical analyses were performed using SAS software, version 9.4, SAS System for Windows, Copyright 2021 (SAS Institute Inc., Cary, NC, USA). A two-sided *p*-value of <0.05 was considered indicative of statistical significance.

## 3. Results

### 3.1. Clinical characteristics

**Figure 1:**
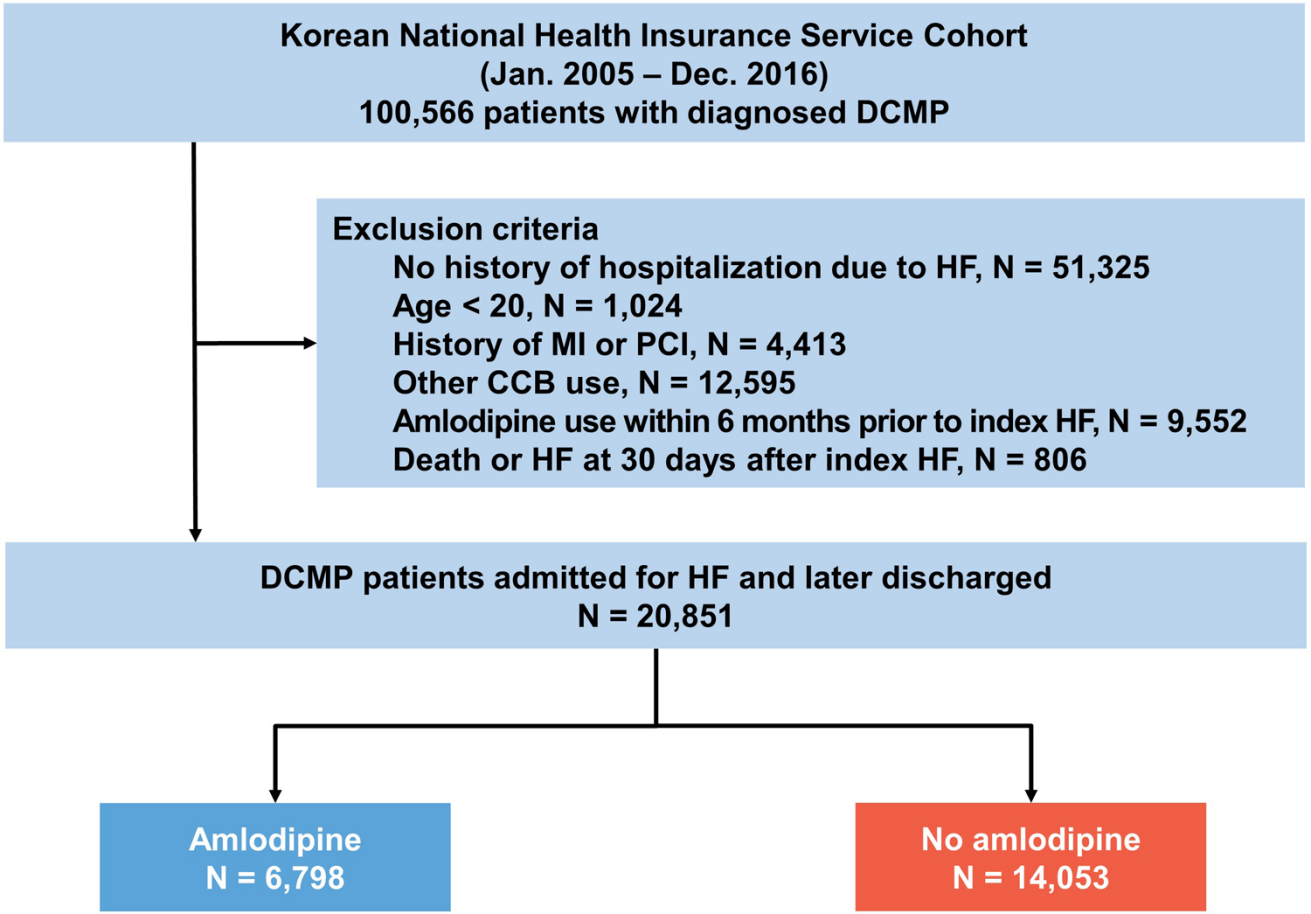
Study flow. The data used in this study were obtained from the Korean national health insurance service cohort. CCB, calcium channel blocker; DCMP, dilated cardiomyopathy; HF, heart failure; MI, myocardial infarction; PCI, percutaneous coronary intervention.

In a cohort of 20,851 patients admitted for HF and subsequently discharged with DCMP, 6,798 patients (32.6%) were prescribed amlodipine (Figure 1 and Table 1). At the index HF event, patients receiving amlodipine were significantly older (63.8 years vs. 58.9 years; *p* < 0.001) and more frequently of female sex (43.7% vs. 41.0%; *p* < 0.001) than those who did not receive amlodipine. Furthermore, the amlodipine group exhibited a higher body mass index (24.6 vs. 23.9 kg/m^2^, *p* < 0.001) and increased systolic (129.6 mmHg vs. 121.0 mmHg, *p* < 0.001) and diastolic blood pressure (BP) (78.6 mmHg vs. 74.5 mmHg, *p* < 0.001) relative to the no-amlodipine group. Regarding comorbidities, the amlodipine group had a higher CCI and higher prevalence of hypertension, diabetes mellitus, and chronic kidney disease, with a lower prevalence of dyslipidemia, valvular heart disease, atrial fibrillation, and malignancy, than the no-amlodipine group. The amlodipine group received fewer prescriptions for all other medications than the no-amlodipine group.

**Table 1:** Baseline clinical characteristics of participants.

Variable	Total (*n* = 20,851)	Amlodipine (*n* = 6,798)	No amlodipine (*n* = 14,053)	*p*
Demographics				
Age, years	60.5 ± 15.6	63.8 ± 14.9	58.9 ± 15.6	<0.001
<40	2,185 (10.5%)	467 (6.9%)	1,718 (12.2%)	<0.001
40–60	7,501 (36.0%)	2,077 (30.6%)	5,424 (38.6%)	<0.001
≥60	11,165 (53.5%)	4,254 (62.6%)	6,911 (49.2%)	<0.001
Female sex	8,737 (41.9%)	2,971 (43.7%)	5,766 (41.0%)	<0.001
Body mass index	24.2 ± 3.8	24.6 ± 4.0	23.9 ± 3.7	<0.001
Blood pressure, mmHg				
Systolic	123.8 ± 16.2	129.6 ± 17.1	121.0 ± 15.0	<0.001
Diastolic	75.8 ± 10.9	78.6 ± 11.5	74.5 ± 10.3	<0.001
Comorbidity				
Hypertension	12,861 (61.7%)	4,520 (66.5%)	8,341 (59.4%)	<0.001
Diabetes mellitus	9,123 (43.8%)	3,032 (44.6%)	6,091 (43.3%)	<0.001
Dyslipidaemia	11,501 (55.2%)	3,515 (51.7%)	7,986 (56.8%)	<0.001
Valvular heart disease	2,321 (11.1%)	504 (7.4%)	1,817 (12.9%)	<0.001
Atrial fibrillation	5,569 (26.7%)	1,182 (17.4%)	4,387 (31.2%)	<0.001
Chronic kidney disease	756 (3.6%)	314 (4.6%)	442 (3.1%)	<0.001
Malignancy	3,854 (18.5%)	1,077 (15.8%)	2,777 (19.8%)	<0.001
Charlson Comorbidity Index	3.0 ± 2.3	2.4 ± 2.4	3.2 ± 2.2	<0.001
Medications				
RAAS inhibitor	11,927 (57.2%)	2,756 (40.5%)	9,171 (65.3%)	<0.001
-ACE inhibitor	6,050 (29.0%)	909 (13.4%)	5,141 (36.6%)	<0.001
-ARB	7,613 (36.5%)	2,107 (31%)	5,506 (39.2%)	<0.001
Beta blocker	12,137 (58.2%)	2,638 (38.8%)	9,499 (67.6%)	<0.001
MR antagonist	7,780 (37.3%)	997 (14.7%)	6,783 (48.3%)	<0.001
Other diuretics	11,312 (54.3%)	2,466 (36.3%)	8,846 (62.9%)	<0.001
Digoxin	4,969 (23.8%)	611 (9.0%)	4,358 (31%)	<0.001
Statin	6,235 (29.9%)	1,808 (26.6%)	4,427 (31.5%)	<0.001
Antiplatelet agent	3,426 (16.4%)	672 (9.9%)	2,754 (19.6%)	<0.001
Anticoagulant	3,298 (15.8%)	583 (8.6%)	2,715 (19.3%)	<0.001

Values are presented as the mean ± standard deviation or *n* (%).

ACE, angiotensin converting enzyme; ARB, angiotensin receptor blockers; HF, heart failure; MR, mineralocorticoid receptor; RAAS, renin–angiotensin–aldosterone system.

### 3.2. Clinical outcomes

**Figure 2:**
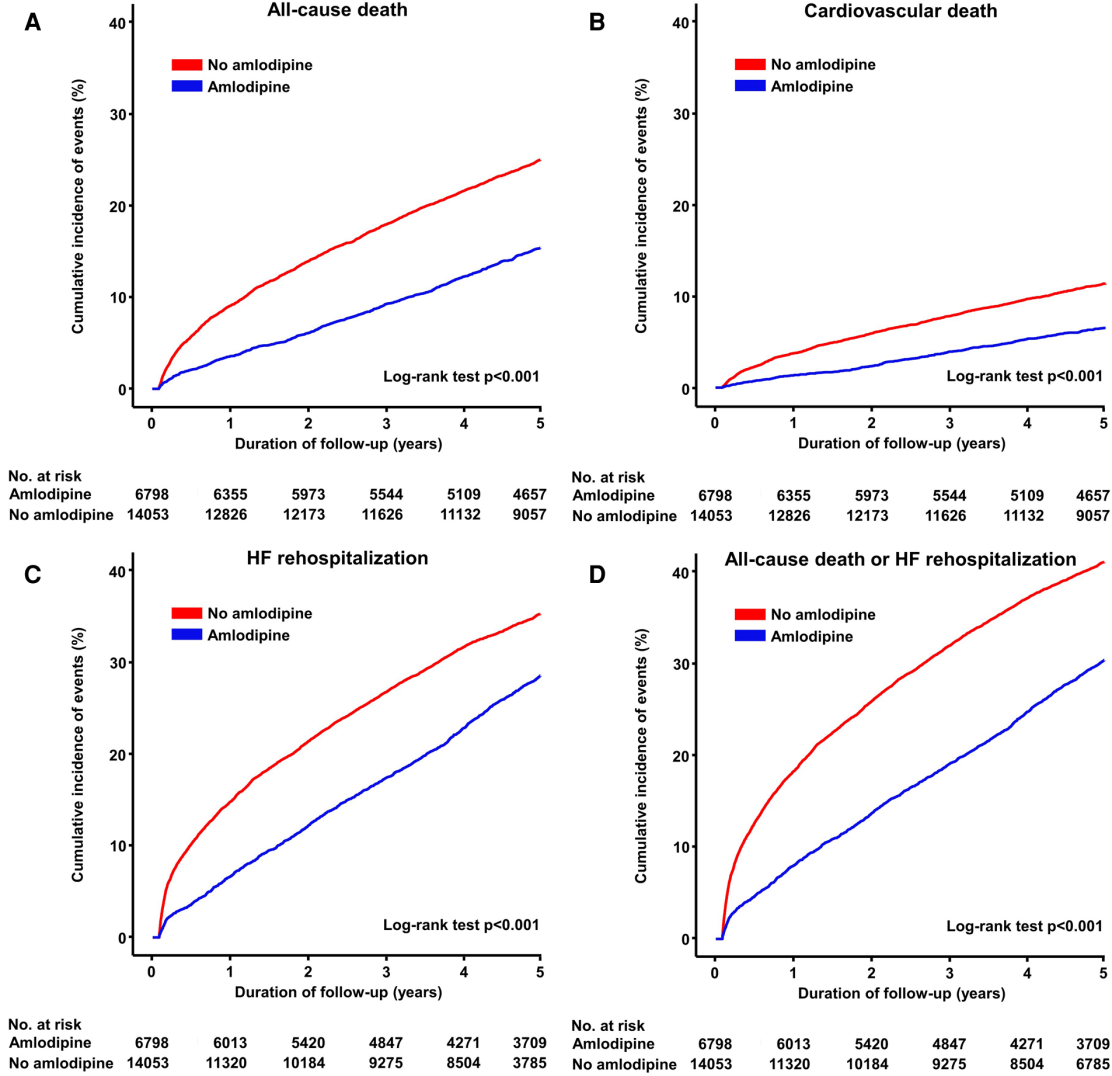
Cumulative incidence of primary and secondary endpoints. Kaplan–Meier curves show the rates of (**A**) all-cause death, (**B**) cardiovascular death, (**C**) HF rehospitalization, and (**D**) composite of all-cause death or HF rehospitalization between amlodipine and no amlodipine use after HF in patients with DCMP. DCMP, dilated cardiomyopathy; HF, heart failure.

During the 5-year follow-up period, 6,798 deaths were reported. Patients receiving amlodipine had a significantly lower risk of all-cause death than those not receiving amlodipine (32.0 vs. 56.6 per 100,000 person-years, adjusted HR: 0.64, 95% CI: 0.59–0.70, *p* < 0.001; Table 2 and Figure 2). The amlodipine group had better clinical outcomes of cardiovascular death and HF rehospitalization (cardiovascular death: 12.2 vs. 22.4 per 100,000 person-years, adjusted HR: 0.71, 95% CI: 0.62–0.81, *p *= 0.001; HF rehospitalization: 66.4 vs. 90.5 per 100,000 person-years, adjusted HR: 0.92, 95% CI: 0.86–0.98, *p *= 0.006). The composite of all-cause death or HF rehospitalization was lower in the amlodipine group than in the no-amlodipine group (81.1 vs. 123.8 per 100,000 person-years, adjusted HR: 0.86, 95% CI: 0.81–0.91, *p* < 0.001). These results were consistent with the propensity score matching and inverse probability weighting adjustments for baseline clinical characteristics (Supplementary Table S2 and Supplementary Figures S1–S3). The sensitivity analysis revealed consistent results in the comparison of clinical outcomes in patients diagnosed with DCM based on ICD (I42) and RID codes (V127) (Supplementary Table S3). We examined the association between amlodipine use and 13 falsification endpoints (Supplementary Table S4). Only one endpoint showed a statistically significant association, whereas the other 12 endpoints did not demonstrate significant relationships (92.3%).

**Table 2:** Clinical outcomes after index HF.

Subjects	*N*	Events	IR*	Unadjusted		Adjusted**		PS-matched		IPW-adjusted	
				HR (95% CI)	*p*	HR (95% CI)	*p*	HR (95% CI)	*p*	HR (95% CI)	*p*
All-cause death											
Amlodipine	6,798	918	32.0	0.56 (0.52–0.61)	<0.001	0.64 (0.59–0.70)	<0.001	0.73 (0.64–0.84)	<0.001	0.58 (0.53–0.63)	<0.001
No amlodipine	14,053	3,350	56.6								
Cardiovascular death											
Amlodipine	6,798	350	12.2	0.54 (0.48–0.61)	<0.001	0.71 (0.62–0.81)	<0.001	0.70 (0.56–0.87)	0.001	0.65 (0.56–0.74)	<0.001
No amlodipine	14,053	1,327	22.4								
HF rehospitalisation											
Amlodipine	6,798	1,712	66.4	0.74 (0.70–0.78)	<0.001	0.92 (0.86–0.98)	0.006	0.91 (0.85–0.96)	0.002	0.85 (0.79–0.90)	<0.001
No amlodipine	14,053	4,460	90.5								
All-cause death or HF rehospitalisation											
Amlodipine	6,798	2,091	81.1	0.66 (0.63–0.69)	<0.001	0.86 (0.81–0.91)	<0.001	0.87 (0.79–0.96)	0.006	0.77 (0.73–0.82)	<0.001
No amlodipine	14,053	6,101	123.8								

* IR: 100 000 person-years.

** Adjusted variables: age, sex, hypertension, diabetes mellitus, dyslipidaemia, valvular heart disease, atrial fibrillation, chronic kidney disease, malignancy, Charlson Comorbidity Index, medications.

CI, confidence interval; HF, heart failure; HR, hazard ratio; IPW, inverse probability weighting; IR, incidence ratio; PS, propensity score.

### 3.3. All-cause death according to subgroup analysis

**Figure 3:**
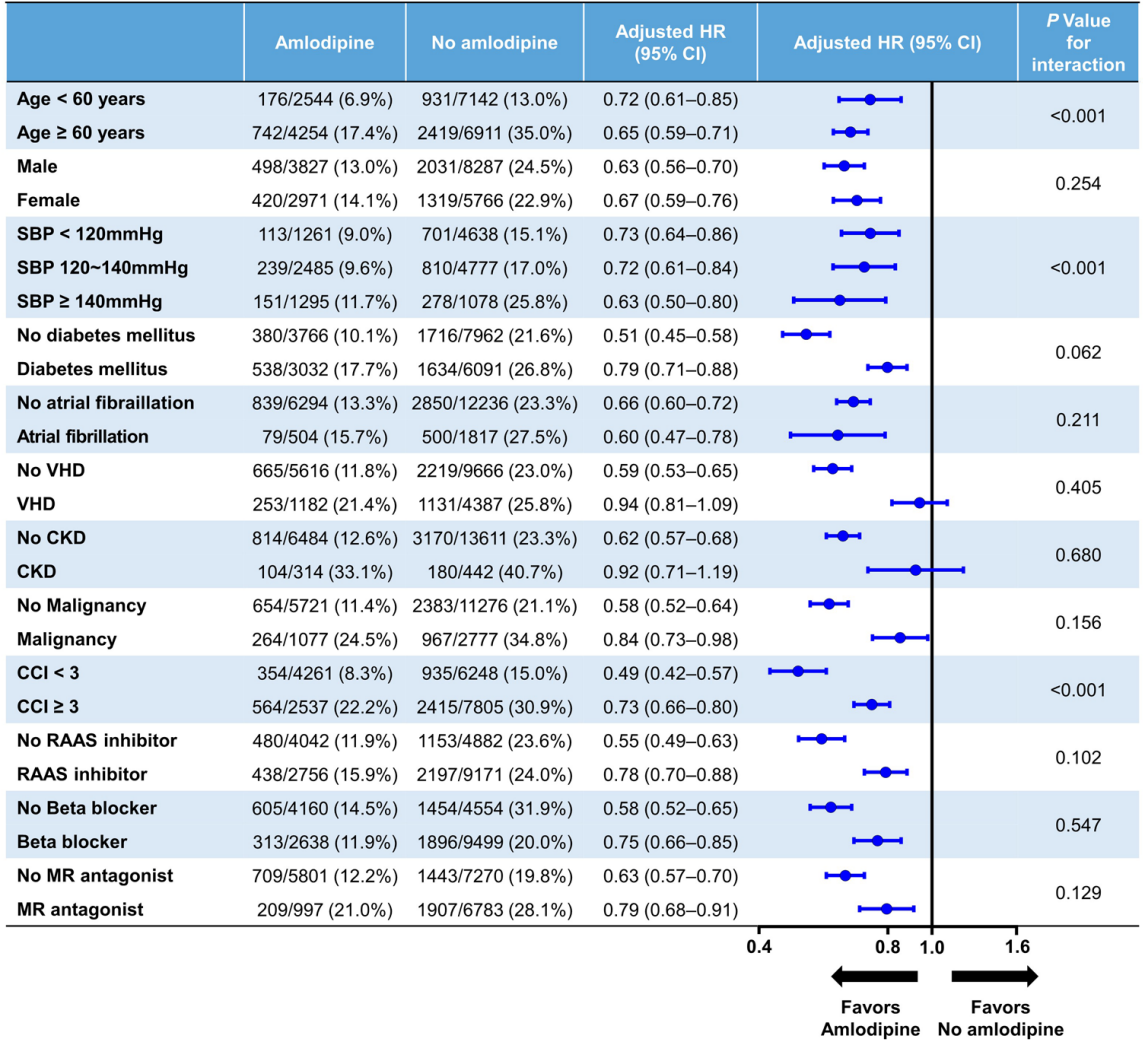
Exploratory subgroup analysis. CCI, Charlson comorbidity index; CI, confidence interval; CKD, chronic kidney disease; HR, hazard ratio; MR, mineralocorticoid receptor; RAAS, renin–angiotensin–aldosterone system; SBP, systolic blood pressure; VHD, valvular heart disease.

A subgroup analysis was conducted based on factors such as age, sex, systolic BP, diabetes, atrial fibrillation, valvular heart disease, chronic kidney disease, malignancy, CCI, and medication use, including renin–angiotensin–aldosterone system inhibitors, beta-blockers, and mineralocorticoid receptor antagonists (Figure 3). The reduced risk of all-cause mortality associated with amlodipine use was consistent across all subgroups. Notably, amlodipine was found to have a significantly greater impact on decreasing all-cause mortality among individuals aged ≥60 years, those with a higher systolic BP, and those with a CCI < 3 (interaction *p* < 0.001).

## 4. Discussion

The current study investigated the clinical efficacy of long-term amlodipine treatment on mortality after HF in patients with DCMP using nationwide medical insurance data in Korea. The findings revealed that amlodipine use was associated with a lower risk of all-cause death than no amlodipine use after hospitalization for HF in patients with DCMP. These favorable outcomes of amlodipine were consistent among different subgroups, particularly among those with an older age, a higher systolic BP, and fewer comorbidities.

This study's results are consistent with those of previous studies that have also demonstrated the beneficial effects of amlodipine in patients with severe chronic HF (10). However, the results of the PRAISE 1 and 2 trials were inconsistent, with the former showing a lower risk of death in severe chronic HF with non-ischemic cardiomyopathy treated with amlodipine compared with placebo, whereas the latter did not show any clinical benefit with amlodipine treatment (11). The authors speculate that these different results may be due to differences in patient characteristics and study design, particularly the proportion of patients with hypertension between the two trials. In the PRAISE 1 trial, the proportion of patients with hypertension was 56%, and the subgroup of patients with hypertension showed a particularly favorable outcome (HR: 0.75, 95% CI: 0.57–0.99), whereas the proportion of patients with hypertension and cardiomyopathy in the PRAISE 2 trial was much lower (17.4%). The proportion of patients with hypertension (61.7%) in the present study was similar to that in the PRAISE 1 trial, which may explain the favorable outcome of amlodipine. These findings are particularly relevant in real-world practice, where amlodipine is mainly used in patients with high BP.

Considering that the role of high BP variability in the prognosis of patients with HF is crucial, studies have shown that increased BP variability not only exacerbates the condition but also leads to adverse clinical outcomes (15). It has been reported that older populations and those with lower CCI scores tend to experience higher BP variability (16, 17). Amlodipine, a calcium channel blocker, has demonstrated beneficial effects on BP variability (18). A better prognosis was observed in patients with a lower CCI and in patients who were older according to the subgroup analysis in the current study. Given the association between higher BP variability and these subgroups, our findings suggest that amlodipine may play a role in improving the outcomes of patients with HF and DCMP by reducing BP variability.

Several potential mechanisms may explain the beneficial effects of amlodipine in patients with HF. First, LV remodeling and diastolic dysfunction are strong predictors of worsening prognosis in patients with DCMP and HF (19–21). Amlodipine has previously shown a positive effect on ventricular remodeling, which is a common feature of HF (22, 23). This medication has been demonstrated to reduce LV mass and volume and improve left diastolic function, which could potentially lead to clinical improvements in HF patient outcomes (24, 25). Second, amlodipine has been reported to have antioxidant properties (26). This characteristic may help protect against oxidative stress and inflammation, both of which are known to play a role in the development and progression of HF. Additionally, amlodipine has been found to inhibit the production of reactive oxygen species and increase the activity of antioxidant enzymes, which could also contribute to its beneficial effects in patients with HF (27, 28). Third, amlodipine has been indicated to have a vasodilatory effect, which could potentially improve myocardial perfusion and oxygen supply, leading to improved clinical outcomes (29, 30). This medication has been revealed to decrease peripheral resistance, increase cardiac output, and reduce myocardial oxygen demand, all of which may contribute to its beneficial effects in patients with HF. Therefore, the various potential mechanisms of action of amlodipine suggest that it may be an effective medication for the treatment of HF.

This study had several strengths. First, evidence of the long-term effectiveness of amlodipine in patients with DCMP after hospitalization for HF was provided. Second, using a nationwide cohort with a large sample size, the statistical power and generalizability of the results have been enhanced. Third, several sensitivity analyses were performed to adjust for potential confounding factors, which helped strengthen the validity of the findings.

This study had several limitations. First, this research was conducted using insurance claim data based on ICD codes, which did not allow for the inclusion of detailed cardiac echocardiogram data such as EF, LV dimensions, and diastolic function. Consequently, the absence of these specific clinical details may impact the depth and specificity of the diagnostic criteria for DCM in our findings. Despite these limitations, our sensitivity analysis using the RID codes showed consistent results, reinforcing the reliability of our findings. Our research, with its large sample size, aims to identify broader epidemiological trends and patterns in a real-world setting, and further research through prospective studies is necessary to clarify the specific benefits of amlodipine. Second, this was an observational study, and the possibility of residual confounding due to unmeasured or unknown confounders could not be excluded. Third, it was conducted in the period before the release of the 2021 European Society of Cardiology HF guidelines, which recommended the use of angiotensin receptor-neprilysin inhibitor and sodium-glucose co-transporter-2 inhibitor as a first-line therapy (6). Hence, the interpretation of the findings should take into account these developments in HF management. Fourth, information on the dosage or duration of amlodipine use, which might have affected the results, was unavailable.

In conclusion, amlodipine use after hospitalization for HF in patients with DCMP in this nationwide cohort study was associated with a lower risk of all-cause death, cardiovascular death, and readmission for HF. Further randomized controlled trials are needed to confirm these findings and explore the optimal use of amlodipine in patients with HF.

## Data Availability

The original contributions presented in the study are included in the article/**Supplementary Material**, further inquiries can be directed to the corresponding author.
