# Supplementary material for: Impact of amlodipine on clinical outcomes for heart failure in patients with dilated cardiomyopathy: a Korean nationwide cohort study
Source: Front Cardiovasc Med. 2023 Nov 17;10:1305824. doi: 10.3389/fcvm.2023.1305824 (PMC10690815; doi:10.3389/fcvm.2023.1305824)

Supplementary Appendix

Supplementary Table S1. Definitions of comorbidities

Supplementary Table S2. Baseline characteristics after propensity-score matching

Supplementary Table S3. Comparison of Clinical Outcomes in Patients Diagnosed with DCM Based on ICD (I42) and rare intractable disease codes (V127)

Supplementary Table S4. Falsification endpoint

Supplementary Figure S1. Cumulative distribution of propensity score before and after inverse probability weighting adjustment

Supplementary Figure S2. Kaplan-Meier curve of the cumulative incidence of clinical outcomes after propensity score matching

Supplementary Figure S3. Kaplan-Meier curve of the cumulative incidence of clinical outcomes after inverse probability weighting adjustment

Supplementary Table S1. Definitions of comorbidities

Diagnosis	ICD-10-CM code and definition
**Hypertension**	I10-I13, I15
**Diabetes mellitus**	E11-E14
**Dyslipidaemia**	E78
**Valvular heart disease**	I05, I06, I07, I08, I34, I35, I36, I37, I38, I39
**Atrial fibrillation**	I48
**Chronic kidney disease**	N18-19
**Malignancy**	C00-C97

Supplementary Table S2. Baseline characteristics after propensity-score matching

Variable	Total (N=4,388)	Amlodipine (n=2,194)	No amlodipine (n=2,194)	p	SMD (%)
Demographics					
Age, years	59.9 ± 15.3	59.6 ± 15.2	60.2 ± 15.4	0.1454	-4.08
<40	475 (10.8%)	249 (11.3%)	226 (10.3%)		
40-60	1,646 (37.5%)	805 (36.7%)	841 (38.3%)		
≥60	2,267 (51.7%)	1,140 (52%)	1,127 (51.4%)		
Female	1,832 (41.8%)	919 (41.9%)	913 (41.6%)	0.8504	-0.55
Comorbidity					
Hypertension	2,866 (65.3%)	1,459 (66.5%)	1,407 (64.1%)	0.105	-4.61
Diabetes mellitus	2,032 (46.3%)	1,058 (48.2%)	974 (44.4%)	0.021	-8.76
Dyslipidaemia	2,653 (60.5%)	1,369 (62.4%)	1,284 (58.5%)	0.010	-8.83
Valvular heart disease	466 (10.6%)	242 (11%)	224 (10.2%)	0.379	-2.73
Atrial fibrillation	1,164 (26.5%)	591 (26.9%)	573 (26.1%)	0.519	-1.94
Chronic kidney disease	195 (4.4%)	103 (4.7%)	92 (4.2%)	0.416	-2.60
Malignancy	875 (19.9%)	439 (20%)	436 (19.9%)	0.908	-0.36
Charlson comorbidity index	3.2 ± 2.3	3.3 ± 2.5	3.1 ± 2.0	0.002	9.08
Medications					

Variable	Total (N=4,388)	Amlodipine (n=2,194)	No amlodipine (n=2,194)	p	SMD (%)
RAAS inhibitor	2,478 (56.5%)	1,232 (56.2%)	1,246 (56.8%)	0.633	1.32
- ACE inhibitor	1,087 (24.8%)	457 (20.8%)	630 (28.7%)		
- ARB	1,694 (38.6%)	928 (42.3%)	766 (34.9%)		
Beta blocker	2,662 (60.7%)	1,374 (62.6%)	1,288 (58.7%)	0.009	-8.20
Spironolactone	1,445 (32.9%)	682 (31.1%)	763 (34.8%)	0.001	8.53
Other diuretics	2,254 (51.4%)	1,139 (51.9%)	1,115 (50.8%)	0.376	-2.27
Digoxin	851 (19.4%)	401 (18.3%)	450 (20.5%)	0.024	5.81
Statin	1,409 (32.1%)	738 (33.6%)	671 (30.6%)	0.025	-6.74
Anti-platelet agent	691 (15.7%)	355 (16.2%)	336 (15.3%)	0.422	-2.47
Anti-coagulant	640 (14.6%)	320 (14.6%)	320 (14.6%)	1.000	0.00

Values are presented as mean ± standard deviation or n (%).

ACE, angiotensin-converting enzyme; ARB, angiotensin receptor blocker; HF, heart failure; RAAS, renin-angiotensin-aldosterone system; SMD, standard mean difference.

Supplementary Table S3. Comparison of Clinical Outcomes in Patients Diagnosed with DCM Based on ICD (I42) and rare intractable disease codes (V127)

Subjects	N	Events	IR*	Unadjusted		Adjusted**	
				HR (95 % CI)	p	HR (95 % CI)	p
All-cause death							
Amlodipine	2263	160	16.2	0.56 (0.47–0.67)	<0.001	0.69 (0.57–0.84)	0.001
No amlodipine	5722	767	28.9				
Cardiovascular death							
Amlodipine	2263	67	6.8	0.44 (0.34–0.56)	<0.001	0.52 (0.39–0.70)	<0.001
No amlodipine	5722	416	15.7				
HF rehospitalisation							
Amlodipine	2263	547	61.8	0.77 (0.80–0.96)	0.008	0.93 (0.86–0.98)	0.018
No amlodipine	5722	1636	70.6				
All-cause death or HF rehospitalisation							
Amlodipine	2263	612	69.1	0.82 (0.75–0.89)	<0.001	0.91 (0.82–0.95)	0.006
No amlodipine	5722	1966	84.9				

*IR: 100 000 person-years

**Adjusted variables: age, sex, hypertension, diabetes mellitus, dyslipidaemia, valvular heart disease, atrial fibrillation, chronic kidney disease, malignancy, Charlson Comorbidity Index, medications.

CI, confidence interval; HF, heart failure; HR, hazard ratio; IR, incidence ratio.

Supplementary Table S4. Falsification endpoint

	Unadjusted		Adjusted*	
Falsification endpoint	HR (95% CI)	p	HR (95% CI)	p
Viral enteritis	0.85 (0.70–1.03)	0.096	0.85 (0.70–1.03)	0.096
Warts	0.75 (0.58–1.08)	0.129	0.78 (0.59–1.04)	0.089
Acute hepatitis A	0.80 (0.58–1.10)	0.165	1.09 (0.77–1.55)	0.639
Viral conjunctivitis	6.05 (0.63–58.17)	0.119	3.45 (0.24–50.35)	0.365
Otitis media	1.02 (0.94–1.11)	0.591	0.96 (0.87–1.06)	0.405
Chronic sinusitis	0.84 (0.78–0.91)	<.0001	0.84 (0.77–0.91)	<0.001
Cellulitis	1.08 (0.98–1.15)	0.1205	1.06 (0.99–1.14)	0.099
Urticaria	0.99 (0.94–1.04)	0.701	0.96 (0.91–1.02)	0.219
Ingrowing nail	1.03 (0.85–1.24)	0.771	1.19 (0.96–1.47)	0.107
Frozen shoulder	1.22 (1.15–1.29)	<.0001	1.00 (0.93–1.07)	0.992
Voice disturbances	0.65 (0.42–1.01)	0.054	0.70 (0.44–1.13)	0.143
Burns	1.07 (0.94–1.22)	0.305	1.11 (0.96–1.29)	0.156
Anaphylaxis / Allergic reaction	0.89 (0.75–1.04)	0.137	0.83 (0.7–1.04)	0.089

*Adjusted variables: age, sex, hypertension, diabetes mellitus, dyslipidaemia, valvular heart disease, atrial fibrillation, chronic kidney disease, malignancy, Charlson comorbidity index, medications.

CI, confidence interval; HR, hazard ratio.

Supplementary Figure S1. Cumulative distribution of propensity score before and after inverse probability weighting adjustment

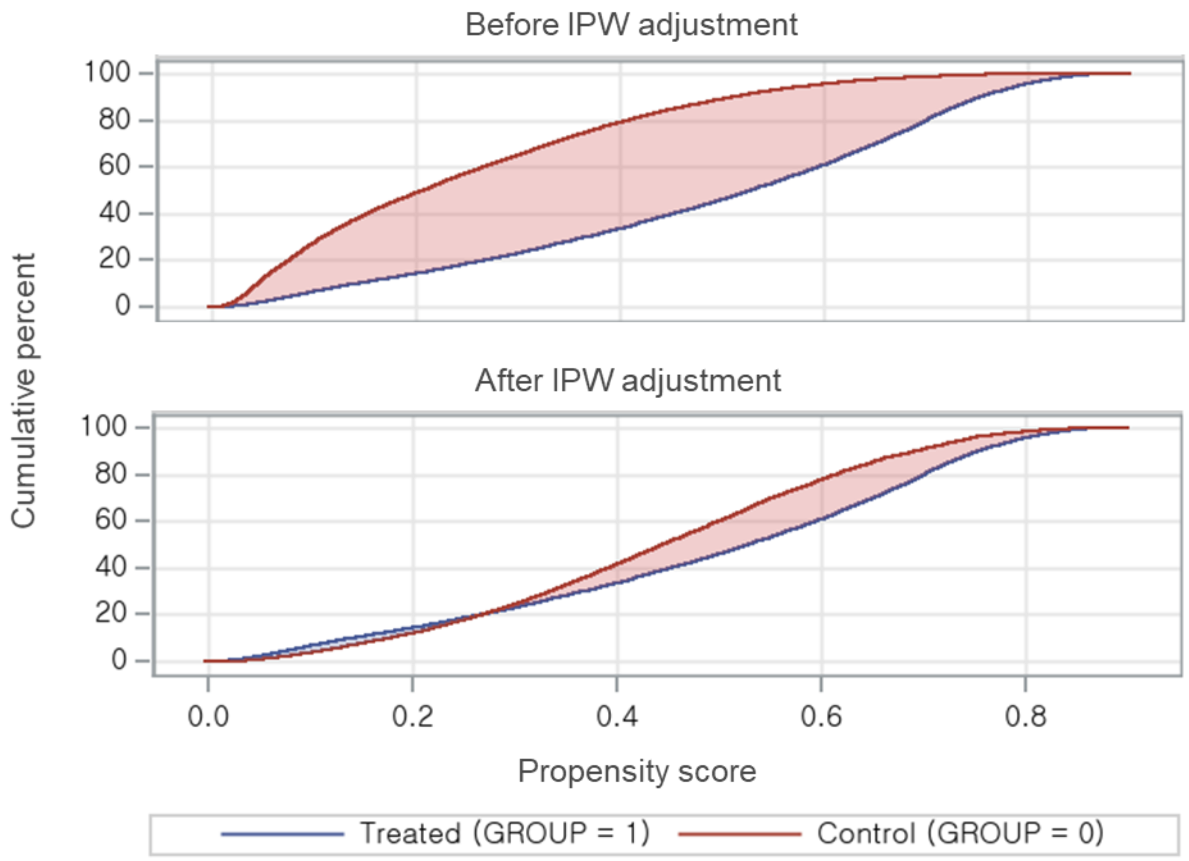

IPW, inverse probability weighting.

Supplementary Figure S2. Kaplan-Meier curve of the cumulative incidence of clinical outcomes after propensity-score matching

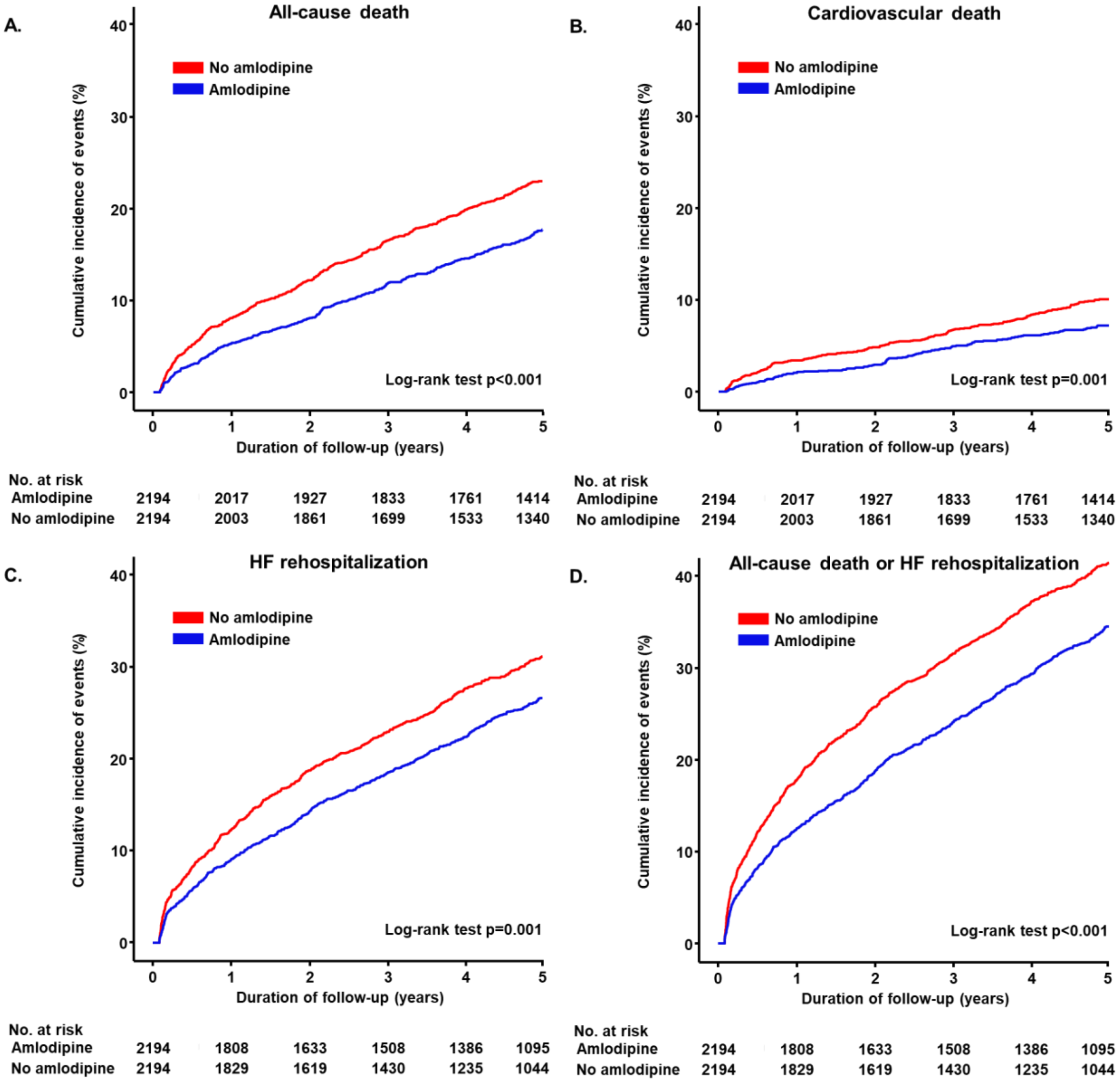

Supplementary Figure S2. Kaplan-Meier curve of the cumulative incidence of clinical outcomes after inverse probability weighting adjustment

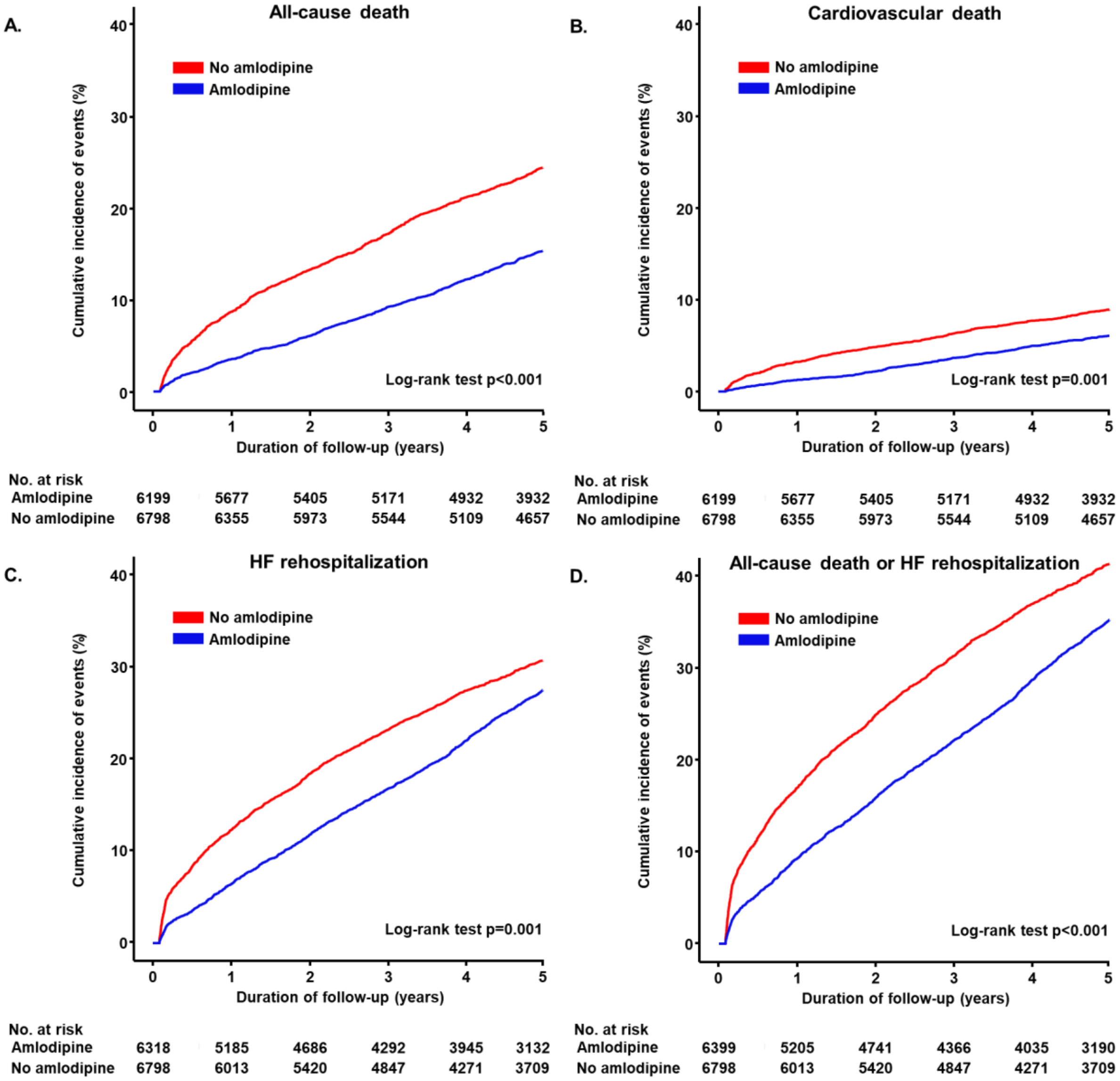

Supplement: Supplementary file 1 [file Datasheet1.pdf]
